# Health Workers' Knowledge and Practice of Developmentally Supportive Care for Premature Infants in Four Ugandan Neonatal Units: A Cross‐Sectional Study

**DOI:** 10.1002/hsr2.71805

**Published:** 2026-02-05

**Authors:** Zelee Hill, Victoria Nakibuuka, Robert Serunjogi, Robert Ssekitoleko, Ritah Nasiima, Sanyu Nalunga‐Atuhe, James Nyonyintono, Albert Kamugisha

**Affiliations:** ^1^ University College London London UK; ^2^ St Francis Nsambya Hospital Kampala Uganda; ^3^ Makerere University Kampala Uganda; ^4^ Kiwoko Hospital Kiwoko Uganda; ^5^ Masaka Regional Referral Hospital Masaka Uganda

**Keywords:** Africa, developmental care, family care, neonatal unit, pre‐term, Uganda

## Abstract

**Background and Aims:**

Neonatal units can be stressful for pre‐term infants at a time of rapid brain growth and plasticity, which may contribute to poorer developmental outcomes. Environmental modification to protect against negative sensory experiences and provide positive caregiver stimuli is the standard of care, but little is known about practices in low‐income settings. We aimed to determine knowledge and practice relating to developmentally‐supportive‐care in Uganda.

**Methods:**

A quantitative survey on knowledge and practice of developmentally‐supportive‐care was conducted with 135 health workers in four neonatal units. In addition, observations of practices and sound measurements were conducted.

**Results:**

Only 36% of respondents reported that stress, and 21% that parental interaction can affect brain development, and knowledge of stress reduction was limited. 84% of respondents reported actions to protect infants from excessive light in their unit, 33% from excessive sound, and 69% from sleep disruption. The main perceived benefit of family involvement in care was to reduce parental stress levels (67%), with infection risk perceived as the main negative (71%). Workers at the largest volume facility had the lowest knowledge and practice, with wide variations across facilities. All units had sound readings over recommended levels.

**Conclusion:**

Knowledge and practice of DSC in this setting were low and needs to be improved to ensure that pre‐terms in this setting both survive and thrive.

## 1 Introduction

There are over 13 million pre‐term births each year, with over a quarter of these births occurring in Sub‐Saharan Africa [1]. With increased rates of facility births in Sub‐Saharan Africa, more of these babies have access to the life‐saving interventions that neonatal units provide. With increasing survival, the longer‐term impacts of prematurity such as the higher risk of neurodevelopment disability (NND) become more prominent, but little research has been conducted in African settings.

Premature delivery disrupts in‐utero developmental processes and increases the risk of complications linked to developmental issues such as brain injury and infections [2]. During this early period, the brain's circuitry is particularly open to the influence of the environment, with chronic or extreme adversity interrupting normal brain development [3, 4]. In the first days and months of life, many pre‐term babies require care in neonatal care units. As this occurs at this time of rapid brain development and plasticity [5, 6], stress in the neonatal unit may contribute to the poorer developmental outcomes seen among those born prematurely [7].

**Figure 1:**
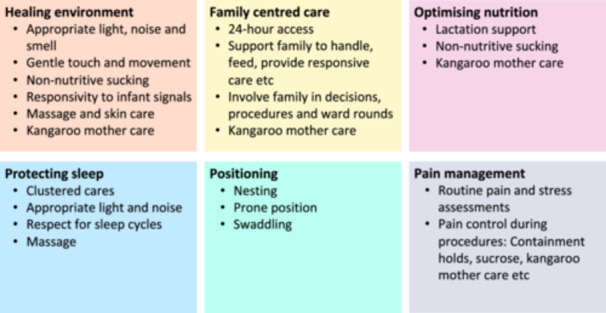
Common components of developmentally supportive care.

Developmentally supportive care (DSC) aims to prevent long‐term developmental consequences associated with the neonatal units' environment by protecting babies from negative sensory experiences and providing positive stimuli through parental touch, smell, and sound [8, 9, 10, 11, 12]. There is no standard package of DSC but common elements are shown in Figure 1 [13]; some components are specific to DSC, but others such as family‐centered‐care and Kangaroo Mother Care (KMC) are important interventions in their own right, that also have impacts beyond neuro‐development [14, 15, 16].

DSC is the standard of care in many high‐income settings [17, 18, 19], with growing evidence of positive impacts [7, 14, 17, 20]. At a global level, DSC is included as a World Health Organisation standard of care for small and sick newborns and as a component of nurturing care [21, 22], but there are no agreed global guidelines around the content or implementation of DSC. Several African countries include DSC in their country‐level guidelines, but the content and detail are very varied. For example, the Clinical Reference Manual for Ethiopia specifies components of DSC in detail, whilst Nigeria's National Guidelines for Comprehensive Newborn Care indicate the need to promote DSC for infants under 1500 g, but give no details of content [23, 24]. Little is known about the feasibility or implementation of these guidelines and where they exist, they are adapted from high‐income settings, and the extent to which DSC can be implemented in low and middle‐income settings is unclear with very limited research in African settings [13, 25].

To add to the sparse knowledge base, we conducted a study in Uganda to determine current DSC knowledge and practice in four neonatal units. Ten percent of births in Uganda are preterm [1], with these babies having substantially higher levels of NDD than their term counterparts (20.4% compared to 7.5%) [26]. The high levels of preterm births and NDD highlight the need for action in this context, but current national guidelines do not include DSC or family involvement in care; research that can feed into guideline development is timely [27].

## 2 Materials and Methods

### 2.1 Study Design and Setting

We conducted a cross‐sectional descriptive survey with health workers, observed practices, and took sound measurements in three urban/peri‐urban and one rural special care neonatal units.

To be eligible for inclusion, hospitals had to have a dedicated clinical area/special care neonatal unit. Four study hospitals that met the criteria were identified during a workshop with health care professionals and stakeholders, and included two government and two private not‐for‐profit mission hospitals. Data were collected between October 22, 2021, and November 11, 2021.

### 2.2 Participants

Two research assistants were based in each facility between 8.00 a.m. and 16.00 p.m. on three consecutive weekdays. All doctors, nurses, midwives, nursing assistants, and any other staff who directly cared for babies were eligible to be interviewed if they worked in the unit during these times. All eligible workers who were on duty during the data collection period were approached at a time they were not busy with clinical work, written informed consent was gained, and an interview time arranged.

### 2.3 Variables and Data Sources

The survey instrument (see Supporting Information S1 and S2) included questions on knowledge of brain development and DSC and current practices. We also asked questions about knowledge of how to establish and maintain breastmilk supply and hygiene (both of staff and parents) as these relate to the feeding and family care components of DSC. The department head and “in charge” were asked additional questions about the unit's structure and policies.

All questions were asked in an open‐ended manner and for questions with more than one possible response, respondents were probed with “anything else” and all responses recorded. The content, response categories, and “correct” knowledge and practice were informed by a literature review of developmental care domains and existing assessment tools [17, 19, 28, 29, 30, 31, 32].

As the identified tools were developed for high‐income settings, we adapted them through team discussion and peer review to make them more relevant to the setting and modified them through a pilot test in one facility. Nine pilot interviews were conducted, and a debrief with data collectors and a review of the responses were used to improve question clarity, response categories, instrument length, and format, and to identify any items with additional training needs. It was not possible to formally test the validity of the instrument. Administration of the final instrument took 15–20 min to administer using surveyCTO.

During the three data collection days, the research assistants also conducted observations lasting between 3 and 5 h around workspace, hygiene practices, noise levels, light exposure, nesting, KMC, and breastfeeding. A structured observation sheet was used to capture this information. The research assistants also took detailed hand‐written notes about what they observed; they photographed and submitted their notes through surveyCTO.

Clearance for the study was gained from each hospital and from the Institutional Review Boards of St. Francis Hospital Nsambya and the Uganda National Council for Science and Technology. At each facility, the study was explained to unit staff in forums such as staff meetings, and staff were provided with a pamphlet explaining the study.

**Biases:** The potential for social desirability bias was reduced by careful design and piloting of the instrument, explanation of the aim of the study to participants, rapport being built over the 3 days of data collection, interviewing in a private space, and through the use of non‐prompted responses. Selection bias was reduced by sampling all eligible health workers present during data collection, however, those working night shifts or outside the 3 days of data collection were not included, which could introduce some bias if the type of health workers varied by shift or time of day.

**Study size:** We calculated that a sample size of 125 was needed based on the assumption that the four units had a total of approximately 250 workers, and knowledge and practice of “correct” DSC was 20%. The assumption of low knowledge and practice was based on the findings of other studies in low and middle‐income settings [33, 34]. Given the facility sizes and shift patterns, we estimated that it would take 3 days to reach the desired sample size.

### 2.4 Quantitative Variables and Statistical Methods

The quantitative data were analyzed in STATA 16 [35], as a whole and by health facility. Given that little is known about the tropic, we a priori decided to report on each item independently rather than generate composite scores. Percentages were used for the descriptive analysis of each item and *χ* ^2^ tests to assess associations or differences between facilities.

There were no refusals or missing data for any variables, but there was considerable use of the “other” category for several variables. “Other” responses were either hand‐coded into existing categories or new categories were generated as needed. Where questions had multiple response categories each category was turned into a dichotomous “yes/no” variable, with the percentage of workers providing that response reported. The written observations were summarized to provide contextual details. In the results, facilities are labeled A–D to maintain anonymity.

## 3 Results

Table 1 shows the characteristics of the 135 respondents, who were predominantly female midwives who had worked in the unit for over a year.

**Table 1:** Characteristics of health workers in four neonatal units in Uganda, 2021 (*N* = 135).

	*n* (%)
Gender	
Female	109 (81)
Male	26 (19)
Age	
20–24	19 (14)
25–29	52 (39)
30–34	37 (27)
34+	27 (20)
Clinical speciality	
Pediatrician	6 (4)
Medical officer/resident	23 (17)
Midwife	48 (36)
Neonatal nurse	21 (16)
Nurse	16 (12)
Student nurse/midwife	12 (9)
Other	9 (7)
Time as a health worker	
< 1 year	20 (15)
1–5 years	48 (36)
6–10 years	39 (29)
> 10 years	28 (21)
Time working in the unit	
< 1 year	39 (29)
1–5 years	73 (54)
> 6 years	23 (17)

The four units had between 12 and 99 beds (See Supporting Information S2: Table S1), and all but one had been over capacity in the last 7 days, with incubator/cot sharing of up to three babies observed in facilities A and D. All but one facility reported an open access policy for parents. All respondents were asked what they felt was the greatest priority for their unit, most frequently reported were issues with staffing, infection control, space, and equipment.

### 3.1 Knowledge and Practice in Relation to Brain Development and Stress

Knowledge of DSC varied (see Table 2), for example, 40% of respondents reported that brain development in the neonatal unit can be supported through KMC, 36% through minimizing stress, and 21% through parental contact. Loud noises, hunger, and painful procedures were reported to cause stress for babies in the neonatal unit by 47%–50% of respondents, but fewer reported other sensory factors as causing stress. Knowledge was very variable across facilities (see Supporting Information S2: Table S2), with facility B (a rural mission hospital) consistently having the highest knowledge and facility A (a large public referral hospital) the lowest.

**Table 2:** Health workers' knowledge and practice of developmental care in four neonatal units in Uganda, 2021: *n* (%) (*N* = 135).

What can support brain development in a neonatal unit	
Breastmilk feeds (optimizing nutrition)*	66 (49)
KMC*	54 (40)
Reduce stress*	49 (36)
Reduce illness and infection	41 (30)
Parental interaction*	29 (21)
Protect sleep*	20 (15)
Reduce noise*	14 (10)
What can increase stress in a neonatal unit	
Loud noise*	67 (50)
Hunger	67 (50)
Painful procedures*	63 (47)
Disturbed sleep*	32 (24)
Bright light*	26 (19)
Lack of parental contact*	21 (16)
Harsh touch*	18 (13)
What can reduce stress in a neonatal unit	
Frequent feeding	67 (50)
KMC*	41 (30)
Parental contact*	40 (30)
Nesting/positioning*	38 (28)
Soft touch*	35 (26)
Reduce light*	23 (17)
Reduce noise*	18 (13)
What is done in the unit to protect babies from light	
Nothing	22 (16)
Shaded windows/drawing curtains*	51 (38)
Eye masks for phototherapy*	51 (38)
Cover incubators*	47 (35)
What is done in the unit to protect babies' sleep	
Nothing	42 (31)
Nesting*	51 (38)
Dark/quiet periods*	40 (30)
Incubator covers*	21 (16)
Clustered cares*	8 (6%)
What is done in the unit to protect babies from sound	
Nothing	90 (67)
Turn off/limit alarms*	30 (22)
No radio*	20 (15)
Talking softly*	12 (9)
What are the benefits of KMC	
Improves attachment/bonding*	113 (84)
Decreases hypothermia*	106 (79)
Helps brain development*	63 (47)
Increases breastfeeding*	57 (42)
Decreases stress in the baby*	42 (31)
Decreases stress in the mother*	39 (29)
Reduces mortality/infection*	21 (16)
Improves weight gain*	18 (13)

* Core developmental care interventions.

Eighty‐four percent of respondents reported that actions were implemented in the unit to protect infants from excessive light (mostly window shades/curtains and phototherapy masks), 69% to protect sleep (mostly nesting), and 33% to protect against excessive sound (mostly turning off/limiting alarms and no radios). Again, there was wide variation by facility: facility B did the most and facility A the least (see Supporting Information S2: Table S2). The large volume facility A had the highest sound readings (89–99 dB), the other units had readings between 62 and 90 dB, and all units had at least one reading over the recommended < 65 dB [36].

Sixty‐four percent of respondents had been trained in KMC, with 21% reporting mortality or infection reduction as a benefit of KMC. KMC was observed in facilities B and D, with one mother practicing KMC during the observation periods in each facility; it was not observed in the other facilities.

### 3.2 Family Involvement and Support

Mothers were most frequently reported as engaging in practical tasks such as feeding, changing nappies, and washing clothes and breastmilk containers (see Table 3). The main perceived benefit of their involvement was reducing parental stress (67%) and increasing attachment and bonding (44%). In the observations, mothers were observed taking babies from cots and incubators to feed them, feeding with nasogastric tubes, and giving oral medicines to their babies without assistance. Infection risk was the most commonly reported negative of their involvement (71%), followed by crowding of the unit (40%). Again, there was a wide variation in responses by facility (see Supporting Information S2: Table S1).

**Table 3:** Health workers' knowledge and practice of family care in four neonatal units in Uganda, 2021: *n* (%) (*N* = 135).

What role do mothers play in the care of their babies in the unit	
Feed baby*	129 (96)
Change nappies*	94 (70)
Wash clothes	65 (48)
Clean area	34 (25)
Bath baby	27 (20)
Check/monitor babies*	21 (16)
KMC*	15 (11)
Comfort/emotionally support baby*	7 (5)
What are the benefits of families helping to care for their babies in the unit	
Reduced parental stress*	90 (67)
Increased attachment/bonding*	60 (44)
Reduces staff workload	51 (38)
Reduces babies stress/crying*	49 (36)
Increases breastfeeding*	37 (28)
Increases parental skills/knowledge*	37 (27)
Improves communication with parents	29 (21)
What are the negatives of families helping to care for babies in the unit	
Infection risk	96 (71)
Crowding	54 (40)
Poor handling of babies	50 (37)
Bothering staff/interfering with care	35 (26)
What information is given to parents	
Status of baby*	111 (82)
Feeding/expressing*	79 (59)
Hygiene	59 (44)
Visiting times	51 (38)
Protocols/rules	15 (11)

* Core developmental care interventions.

Eighty two percent of respondents reported that parents were given information about the status of the baby, but support for caring activities was limited. No workers were observed being rude or shouting at mothers.

### 3.3 Knowledge of Establishing and Maintaining Breastmilk Supply

Drinking water and breast massage were most frequently reported as a means to stimulate breastmilk supply. Expressing early and often, KMC/contact, counseling/support, rest, and decreasing maternal stress were reported by less than 25% of respondents, and non‐nutritive sucking was not reported at all (see Table 4). Facility B had the highest knowledge and Facility A the lowest (see Supporting Information S2: Table S2).

**Table 4:** Health workers knowledge of establishing and maintaining breastmilk supply in four neonatal units in Uganda, 2021 *n* (%) (*N* = 135).

When should a mother of a pre‐term start expressing milk	
Immediately/within 6 h*	55 (41)
Day 1–3	47 (35)
Depends on the baby's condition	23 (17)
How many times in a 24 h period should a mother of a pre‐term express milk	
1–2 times	7 (5)
3–6 times	13 (10)
7 or more*	109 (81)
What can be done to help a mother stimulate her milk supply	
Drink water/fluids	82 (61)
Breast massage	63 (47)
Express/breastfeed often*	43 (32)
Express early*	32 (24)
KMC/contact with baby*	25 (19)
Reduce maternal stress*	22 (16)
Adequate diet	19 (14)
Counsel/support mother*	14 (10)
Are mothers provided with any equipment or containers for expressing	
No	36 (27)
Bowls/cups	66 (49)
Bottles	62 (46)
Syringes	39 (29)
What are the most frequent ways equipment and containers for expressing are cleaned	
Mother washes	89 (66)
Staff washes	59 (44)
Milton tablets	24 (18)
Sterilized	16 (12)

* Core developmental care interventions.

### 3.4 Hygiene Practices

Hand washing varied across the facilities, for example, in the observations, staff and mothers in Facility C were consistent with hand washing and in cleaning equipment between babies, but this was not the case in the other facilities. Facility D did not have a sink in the unit, but some health staff and mothers were observed using hand sanitizer.

## 4 Discussion

Knowledge of developmentally‐supportive‐care varied, with some gaps in knowledge of the impact of stress and parental contact on brain development. Half of the respondents reported that loud noises, hunger, and painful procedures could cause stress, but knowledge of other causes and ways to reduce stress was lower. Few respondents reported activities to protect babies from excessive sound or that actions were taken to protect sleep, such as nesting, quiet times, or clustered cares. Actions to protect babies from light were more common and included shaded windows, phototherapy masks, and incubator covers. All the special care neonatal units had sound readings over the recommended < 65 dB. To implement developmentally‐supportive‐care health workers will require new knowledge and skills, with perceptions and knowledge associated with the implementation of DSC in other settings [37, 38].

Knowledge and practice varied significantly across the four facilities, with the large volume unit (A) having the lowest knowledge, poorest practices, and the highest sound readings. These different starting positions need to be taken into account when designing and implementing DSC interventions. More research is needed on what causes these differences, but wide variations in care, resources, skills, motivation and outcomes has been reported as common in low and middle‐income health settings [39].

Family‐centered care and KMC are an integral part of DSC and key interventions in their own right [16]. Mothers were reported to engage in practical tasks, and we observed that they had autonomy in relation to handling and feeding their babies. Health workers were never observed being rude or shouting at mothers. These positive practices are encouraging and can be built on. Health workers mainly perceived the benefits of parental involvement as reducing parental stress rather than improving outcomes for the infant and family. Knowledge of benefits of KMC on development, stress, and milk supply needs to be highlighted to health workers as does its impact on mortality, whilst KMC is viewed as predominantly an intervention for attachment and hypothermia implementation may be low. Concerns about parents as a source of infection need to be addressed; this is a common rationale for limiting parental contact in other settings, despite evidence that it is not associated with increased infections [14, 40]. In some settings, family members are being used to fill staffing gaps by, for example, taking temperatures and weights, feeding, and being taught danger signs [39]. We saw some evidence of this occurring in the study facilities in relation to feeding and the provision of oral medicines, but family centered care should be more than using parents as an extra pair of hands, but a “re‐positioning of power” [16].

Three of the study facilities had been over capacity for most days in the last week, and respondents reported staffing and space as priorities for change. In the observations cot/incubator sharing, which can be common in low and middle‐income settings [41], was observed in two facilities. Issues with the structural environment can be a major implementation barrier to DSC through high workloads, lack of time, lack of equipment (e.g., linen for nesting), and overcrowding (e.g., increasing noise levels) [37, 38, 42, 43]. The need for structural changes has been highlighted in the “new vision” for the implementation of KMC and family centered care [16, 44], and DSC should be included when rethinking the structure of neonatal units as we strive to reach the goals of 80% of districts having at least one level‐2 inpatient unit for small and sick newborns by 2025 [45].

Studies have found that in‐hospital neonatal mortality rates in Uganda range from 2.8% to 22.5% [41], with the need for better management and prevention of hypothermia and respiratory distress, greater implementation of KMC, early initiation of breastfeeding, and improved staffing, space, and equipment [46, 47, 48, 49]. Efforts to improve DSC in Uganda must go hand in hand with quality improvements, which have been shown to be feasible and effective in reducing mortality in this setting [39, 50]. This study was not designed to measure quality gaps, but we identified the need to improve hand washing and equipment cleaning (included utensils for collecting and storing expressed breastmilk), reduce overcrowding, and improve the provision of KMC.

The observations and health worker responses triangulated well, except for around KMC, but health worker responses may have been influenced by social desirability bias. It was not possible to test the validity of the instrument – this is an important limitation given that the instrument was adapted from high‐income settings. Practices may have been modified under observation, and we only observed if hands were washed rather than how it was done. We were unable to interview and observe during the night shift; this may limit the generalizability of our findings, particularly if night staff operate under different resource constraints, supervision levels, or routines that influence developmental care practices. We were only able to include four facilities, and findings might be different in other facilities. Data were collected during the COVID‐19 pandemic, although not during a period with restrictions on movement or access to the clinic, but this might also have affected practices.

The potential for social desirability bias was reduced by careful design and piloting of the instrument, explanation of the aim of the study to participants, rapport being built over the 3 days of data collection, interviewing in a private space and through the use of non‐prompted responses. Selection bias was reduced by sampling all eligible health workers present during data collection; however, those working night shifts or outside the 3 days of data collection were not included, which could introduce some bias if the type of health workers varied by shift or time of day.

## 5 Conclusion

This study found substantial gaps in health worker knowledge and practice related to developmentally supportive care, particularly in stress reduction, sensory protection, and the role of parental contact. Facility‐level variation was marked, with the highest‐volume unit showing the lowest knowledge and poorest practices. These findings underscore the need to integrate DSC into Uganda's national newborn care guidelines, in line with WHO standards for small and sick newborns and the Nurturing Care Framework, which emphasize responsive caregiving and family involvement as essential components of early childhood development, and improvements to the structural environment to support quality care for preterm infants.

## Author Contributions

**Zelee Hill:** conceptualization, methodology, formal analysis, supervision, project administration, writing – original draft, funding acquisition, investigation, validation, visualization. **Victoria Nakibuuka:** conceptualization, methodology, supervision, writing – review and editing, investigation, funding acquisition, resources, project administration. **Robert Serunjogi:** methodology, writing – review and editing. **Robert Ssekitoleko:** methodology, investigation, writing – review and editing, project administration. **Ritah Nasiima:** writing – review and editing, investigation, methodology. **Sanyu Nalunga‐Atuhe:** investigation, methodology, writing – review and editing. **James Nyonyintono:** methodology, writing – review and editing. **Albert Kamugisha:** methodology, writing – review and editing.

## Ethics Statement

Approval was gained from each hospital and from the Institutional Review Boards of St. Francis Hospital Nsambya and the Uganda National Council for Science and Technology.

## Consent

Signed informed consent was obtained from all participants (IRB number: HS1254ES).

## Conflicts of Interest

The authors declare no conflicts of interest.

## Transparency Statement

The lead author, Zelee Hill, affirms that this manuscript is an honest, accurate, and transparent account of the study being reported; that no important aspects of the study have been omitted; and that any discrepancies from the study as planned (and, if relevant, registered) have been explained.

## Supporting information

Staff survey developmental care clean 29 09 21(1).

**Supplementary Table S1:** Facility characteristics. **Supplementary Table S2:** Knowledge and practice of developmental care by facility % (n) (N=135) Chi‐squared test used to assess associations or differences between facilities (** P<.001 *P<.05)

## Data Availability

Anonymized data will be made available on request from the corresponding author: z.hill@ucl.ac.uk.
