# Supplementary material for: Health Workers' Knowledge and Practice of Developmentally Supportive Care for Premature Infants in Four Ugandan Neonatal Units: A Cross‐Sectional Study
Source: Health Sci Rep. 2026 Feb 5;9(2):e71805. doi: 10.1002/hsr2.71805 (PMC12876464; doi:10.1002/hsr2.71805)
Supplement: Supplementary file 1 — Staff survey developmental care clean 29 09 21(1). [file HSR2-9-e71805-s001.pdf]

UGANDAN DEVELOPMENTAL CARE SURVEY 2021

SECTION 1. IDENTIFICATION

101	Interviewer code	|___||___|
102	Date of Interview [DD/MM/YY]	|___||___| |___||___||___||___|
103	Facility	1. Kawempe 3. Nsambya<br>2. Kiwoko 4. Naguru

SECTION 2. BACKGROUND

Interviewer: Thank you for agreeing to respond to this survey. I would first like to ask you about background.

201	Sex of respondent	1. Male 2. Female
202	How old are you?	|___||___| Years
203	What is your clinical speciality?	1. Neonatologist 5. Nursing assistant<br>2. Paediatrician 6. Midwife<br>3. Medical officer 7. Other: ___________<br>4. Neonatal nurse
204	How long have you been a health worker?	1. Less than one year 3. 6-10 years<br>2. 1-5 years 4. Over ten years
205	How long have you worked in this NICU?	1. Less than one year 3. 6-10 years<br>2. 1-5 years 4. Over ten years
206	Do you have a position of responsibility in this NICU?	1. Head of NICU. 3. None/other **(Skip to section 4)**<br>2. In-charge

SECTION 3. FACILITY AND STAFF

Interviewer: Now I would like to ask you some questions about how the NICU is structured

301	In the last 24 hours, how many babies have been in the NICU *[99 NK]*	|___||___| babies
302	In the last 24 hours, how many doctors were working in the NICU? *[99 NK]*	|___||___| doctors
303	In the last 24 hours, how many nurses/ midwives were working in the NICU? *[99 NK]*	|___||___| nurses/midwives
304	What is the bed capacity of your NICU?	|___||___| beds
305	In the last 7 days, how many days were you over this capacity? *[99 NK]*	|___| days
306	What happens mopst often when you reach capacity?	1. Admitted anyway to NICU 4. Don't know<br>2. Referred to another facility 5. Never over capacity/no standard<br>3. Sent home/not admitted 6. Other: ___________
307	Do you have a dedicated space for parents to do KMC?	1. Yes 2. No
308	Are any of the NICU staff trained on how to support parents to do KMC?	1. Yes 2. No

309	Has any parent done KMC in the last 24 hours?	1. Yes	2. No
310	When are parents allowed into the NICU? *Do not prompt except: Anything else?*	1. Feeding times<br>2. Open access<br>3. Visiting times	4. Don't know<br>5. Other_________
311	Where do mothers most often sleep when their babies are admitted?	1. Dormitory<br>2. Ward<br>3. Corridors	4. At home<br>5. Don't know<br>6. Other________
312	What do mothers most often for food when their babies are admitted?	1. Food provided<br>2. Cook in the facility<br>3. Buy/bring from outside	4. Don't know<br>5. Other________
313	Please tell me if the following equipment are available and functional in your NICU in the last 24 hours?		
	Baby weighing scales	1. Yes	2. No
	Thermometers	1. Yes	2. No
	Stethescopes	1. Yes	2. No
	Pulse oximeters	1. Yes	2. No
	Non invasive BP monitors	1. Yes	2. No
	Largyoscopes	1. Yes	2. No
	Suction machines	1. Yes	2. No
	Ambu bag and mask sizes 0 & 1	1. Yes	2. No
	Newborn Nasal Gastric tubes	1. Yes	2. No
	Newborn canulas	1. Yes	2. No
	Sterilizer/autoclaves	1. Yes	2. No
	Sharp disposal	1. Yes	2. No
	Fridge	1. Yes	2. No
	Soap	1. Yes	2. No
	Disinfectant	1. Yes	2. No
	Gloves	1. Yes	2. No
	Tetracycline ointment	1. Yes	2. No
	Gentamycin	1. Yes	2. No
	Surfacatant	1. Yes	2. No
	IV fluids	1. Yes	2. No
	CPAP	1. Yes	2. No
	Ventilator	1. Yes	2. No
	Oxygen	1. Yes	2. No
	Blended Oxygen	1. Yes	2. No
314	Who is responsible for cleaning the NICU?	1. No-one/ad-hoc<br>2. Nurses	3. Cleaning staff<br>4. Other________________

315	How often is cleaning done?	1. As needed<br>2. Daily<br>3. Twice daily<br>4. Other________________
	Interviewer: Now I would like to ask you some questions about how the COVID-19 pandemic has affected the NICU	
316	Overall how has the COVID-19 pandemic impacted staffing levels in the NICU?<br><br>*Do not prompt except: Anything else?*	1. No impact<br>2. Fewer staff<br>3. Shorter working days<br>4. Longer shifts<br>5. Don't know<br>6. Other: ___________
317	Overall how has the COVID-19 pandemic impacted the number of babies admitted?	1. No impact<br>2. Fewer babies<br>3. More babies<br>4. Don't know<br>5. Other: ___________
318	Overall how has covid impacted parental access to the NICU?<br><br>*Do not prompt except: Anything else?*	1. No impact<br>2. Reduced access<br>4. Don't know<br>5. Other: ___________
319	Has covid had any other impact on the NICU?<br><br>*Do not prompt except: Anything else?*	1. Yes. Specify:___________<br>2. No<br>4. Don't know

SECTION 4. KNOWLEDGE OF DEVELOPMENTAL CARE

Interviewer: Now I would like to ask you some questions about what you know about know and think

401	What is the most important priority for improvement in this NICU?<br>*Only once answer allowed*	1. Infection control<br>2. Breastfeeding rates<br>3. Staff numbers<br>4. Space<br>5. Electricity<br>6. Water<br>7. Equipment<br>8. Other_____________
402	How can the brain development of a preterm in a NICU be improved?<br>*Do not prompt except: Anything else?*	1. Reduce stress<br>2. KMC<br>3. Maternal interaction<br>4. Protect sleep<br>5. Breastmilk feeds<br>6. Reduce illness and infection<br>7. Don't know<br>8. Other_____________
403	What may cause a baby to have increased stress in a NICU?<br>*Do not prompt except: Anything else?*	1. Loud noise<br>2. Bright light<br>3. Painful procedures<br>4. Disturbed sleep<br>5. Lack of parental contact<br>6. Harsh touch<br>7. Hunger<br>8. Don't know<br>9. Other_____________
404	What can be done to reduce newborn stress in a NICU?<br>*Do not prompt except: Anything else?*	1. KMC<br>2. No direct sunlight<br>3. Sucrose<br>4. Nesting<br>5. Parental contact<br>6. Soft touch<br>7. Frequent feeding<br>8. Don't know<br>9. Other_____________
405	What can be done to make a baby feel more comfortable and safe in an incubator/cot?<br>*Do not prompt except: Anything else?*	1. Nesting<br>2. Clothe from mother<br>3. Containment holding<br>4. Change babies position<br>5. Don't know<br>6. Other_____________
406	When should the mother of a preterm start expressing milk?	1. Within 6 hours<br>2. Day1-3<br>3. > day 3<br>4. When the infant is stable<br>5. Don't know<br>6. Other____________

407	How many times in a 24 hour period should the mother of a preterm express milk in the first few days after birth?	1. 1-2 times<br>2. 3-4 times<br>3. 5-6 times	4. 7 or more times<br>5. Don't know<br>6. Other___________
408	What can be done to help a mother stimulate their milk supply?<br>*Do not prompt except: Anything else?*	1. KMC/contact with baby<br>2. Breast massage<br>3. Express early<br>4. Express often	5. Drink water<br>7. Herbal medicine<br>8. Don't know<br>9. Other___________
409	At what age can premature babies start to suck?	1. <32 weeks<br>2. 32-34 weeks	3. >34 weeks<br>4. Don't know
410	What are the benefits of families helping to care for babies in the NICU?<br>*Do not prompt except: Anything else?*	1. Extra pair of hands<br>2. Increases attachment<br>3. Baby cries less<br>4. Increases breastfeeding<br>5. Reduces newborn stress	6. Reduces parental stress<br>7. Increases skills and knowledge<br>8. Increases HW communication<br>9. Don't know<br>10. Other____________
411	What are the negatives of families helping to care for babies in the NICU?<br>*Do not prompt except: Anything else?*	1. Infection risk<br>2. Poor handling practices<br>3. Crowding	4. Bothering staff<br>5. Don't know<br>6. Other___________
412	Have you received training in KMC?	1. Yes	2. No
413	What is Kangaroo mother care?<br>*Circle all mentioned*	1. Kangaroo position<br>2. Kangaroo nutrition<br>3. Early discharge	4. Don't know<br>5. Other___________
414	When should KMC begin?	1. As soon as possible<br>2. When baby is stable<br>3. When baby is cold	4. When there is no incubator<br>5. Don't know<br>6. Other____________
415	How often should KMC be done in a 24 hour period?	1. As much as possible<br>2. 7-12 hours a day<br>3. 3-6 hours a day	4. 0-2 hours a day<br>5. Don't know<br>6. Other____________
416	What are the benefits of KMC?<br>*Do not prompt except: Anything else?*	1. Increases breastfeeding<br>2. Decreases hypothermia<br>3. Decreases mortality<br>4. Reduces newborn stress<br>5. Reduces parental stress	6. Increases attachment<br>7. Improves brain development<br>8. Earlier discharge<br>9. Don't know<br>10. Other____________
**SECTION 5. PRACTICE**			
Interviewer: Now I would like to ask you some questions about what happens in this NICU			
501	What role do mothers play in the care of their babies in the NICU?<br>*Do not prompt except: Anything else?*	1. Feeding baby<br>2. Bathing baby<br>3. Washing clothes<br>4. Changing nappies	5. Cleaning<br>6. Giving medicines<br>7. Other________<br>8. Don't know
502	Is any information given to parents when the baby is admitted	1. Yes<br>2. No **(Skip to 504)**	3. Don't know **(Skip to 504)**
503	What information is given?<br>*Do not prompt except: Anything else?*	1. Fee structure<br>2. Visiting times	6. Hygiene<br>7. Don't know

		3. Feeding/expressing<br>4. KMC<br>5. Status of baby	8. Other________<br>9. Don't know
504	Is any support given to mothers around expressing milk?	1. Yes<br>2. No **(Skip to 506)**	3. Don't know **(Skip to 506)**
505	What support is given?<br>*Do not prompt except: Anything else?*	1. When to start<br>2. How often to express<br>3. Expressing techniques<br>4. Provision of containers	5. Importance of KMC<br>6. Don't know<br>7. Other________
506	What happens if a mother does not have enough breast milk?<br>*Do not prompt except: Anything else?*	1. Baby given dextrose<br>2. Baby given formula<br>3. KMC encouraged<br>4. Latch/position checked	5. Expression frequency checked<br>6. Nothing<br>7. Don't know<br>8. Other________
507	Are mothers provided with any equipment or containers for expression?	1. None<br>2. Syringes<br>3. Bottles	4. Bowls<br>5. Don't know<br>6. Other________
508	What is the most frequent way equipment or containers used for expressing are cleaned?	1. Sterilized<br>2. Staff washes<br>3. Mum washes in NICU<br>4. Mum washes elsewhere	5. Don't know<br>6. No equipment or containers<br>7. Other________
509	Has KMC been done in the facility in the last 7 days?	1. Yes<br>2. No **(Skip to 512)**	
510	Where is KMC most often done	1. Designated KMC area<br>2. Main ward<br>3. Don't know	4. Other________
511	Do most parents practice KMC in this facility?	1. Yes **(Skip to 513)**<br>2. No	3. Don't know
512	What are the reasons KMC is not practiced more?	1. Lack of space<br>2. Mothers too busy<br>3. Mothers refuse<br>4. Lack of privacy	5. Lack of chairs/beds<br>6. Other________<br>7. Don't know
513	Is anything done to protect/aid babies sleep in the NICU?	1. Yes<br>2. No **(Skip to 515)**	3. Don't know **(Skip to 515)**
514	If yes, what is done?<br>*Do not prompt except: Anything else?*	1. Dark/quiet periods<br>2. Clustered cares<br>3. Nesting<br>4. Incubator covers	5. Cares given when baby aroused<br>6. Don't know<br>7. Other____________
515	Is anything done to protect babies from being exposed to too much light?	1. Yes<br>2. No **(Skip to 517)**	3. Don't know **(Skip to 517)**
516	What is done<br>*Do not prompt except: Anything else?*	1. Shaded windows<br>2. Cots not in the sun<br>3. Eye masks<br>4. Incubator covers	5. Don't know<br>6. Other____________

517	Is anything done to protect babies from excess sound in the NICU?	1. Yes<br>2. No **(Skip to 519)**<br>3. Don't know **(Skip to 519)**
518	What is done?<br>*Do not prompt except: Anything else?*	1. Soft talking<br>2. Turn of alarms quickly<br>3. Incubator covers<br>4. Low phone/pager rings<br>5. Padded bins/doors<br>6. No radios<br>7. Nothing placed on incubator<br>8. Don't know<br>9. Other____________
519	At what times are staff able to wash/sanitise their hands in the NICU?<br>*Do not prompt except: Anything else?*	1. On entering the NICU<br>2. Before examining baby<br>3. After nappy change<br>4. Before feeding a baby<br>5. Don't know<br>6. Other____________

Interviewer: Thankyou for answering this survey; is there anything else you think would be useful for us to know about this NICU or your work?
