# Supplementary material for: Health Workers' Knowledge and Practice of Developmentally Supportive Care for Premature Infants in Four Ugandan Neonatal Units: A Cross‐Sectional Study
Source: Health Sci Rep. 2026 Feb 5;9(2):e71805. doi: 10.1002/hsr2.71805 (PMC12876464; doi:10.1002/hsr2.71805)
Supplement: Supplementary file 2 — Supplementary Table S1: Facility characteristics. Supplementary Table S2: Knowledge and practice of developmental care by facility % (n) (N=135) Chi‐squared test used to assess associations or differences between facilities (** P<.001 *P<.05) [file HSR2-9-e71805-s002.docx]

**Supplementary Table S1: Facility characteristics**

|  | **Facility A** | **Facility B** | **Facility C** | **Facility D** |
| --- | --- | --- | --- | --- |
| Type of facility | Public referral hospital (urban) | Mission hospital (rural) | Mission referral hospital (urban) | Public general hospital (urban) |
| Number of babies in unit in the last 24 hours | 90 | 45 | 12 | 15 |
| Bed capacity | 99 | 38 | 20 | 12 |
| Number of days over capacity in last 7 days | 4 | 7 | 0 | 7 |
| Doctors working in the last 24 hours | 10 | 3 | 5 | 3 |
| Nurses/midwives working in the last 24 hours | 5 | 17 | 5 | 9 |
| When are parents allowed in the unit | Feeding | Open access | Open access | Open access |
| Greatest priority for the unit n (%) | | | | |
| Infection control  Staffing  Space  Equipment  Other | 8 (24)  11 (33)  2 (6)  4 (12)  8 (24) | 3 (8)  19 (53)  10 (28)  3 (8)  1 (3) | 11 (31)  5 (14)  3 (9)  10 (29)  6 (17) | 0 (0)  1 (3)  10 (32)  19 (61)  1 (3) |

**Supplementary Table S2: Knowledge and practice of developmental care by facility** % (n) (N=135) Chi-squared test used to assess associations or differences between facilities (** P<.001 *P<.05)

|  | **Facility A**  N=33 | **Facility B**  N=36 | **Facility C**  N=35 | **Facility D**  N= 31 |
| --- | --- | --- | --- | --- |
| **Knowledge and practice of developmental care:** | | | | |
| How can the brain development of a pre-term in a Newborn Unit be improved? | | | | |
| Breastmilk feeds** | 10 (30) | 12 (33) | 26 (74) | 18 (58) |
| Practice KMC** | 7 (21) | 16 (44) | 27 (77) | 4 (13) |
| Reduce stress** | 3 (9) | 22 (61) | 7 (20) | 17 (55) |
| Reduce illness and infection** | 3 (9) | 14 (39) | 11 (31) | 13 (42) |
| Protect sleep** | 2 (6) | 17 (47) | 0 (0) | 1 (3) |
| Maternal interaction** | 3 (9) | 13 (36) | 3 (9) | 10 (32) |
| Reduce noise** | 3 (9) | 10 (28) | 0 (0) | 1 (3) |
| What may cause a baby to have increased stress in a newborn unit? | | | | |
| Loud noise** | 9 (27) | 32 (89) | (13) 37 | 13 (42) |
| Hunger** | 10 (30) | 16 (44) | (21) 60 | 20 (65) |
| Painful procedures | 10 (30) | 22 (61) | 15 (43) | 16 (52) |
| Disturbed sleep** | 1 (3) | 19 (53) | 11 (31) | 1 (3) |
| Bright light | 5 (15) | 12 (33) | 5 (14) | 4 (13) |
| Lack of parental contact** | 0 (0) | 8 (22) | 4 (11) | 9 (29) |
| Harsh touch** | 2 (6) | 10 (28) | 4 (11) | 2 (6) |
| What can be done to reduce newborn stress in a newborn unit | | | | |
| Frequent feeding | 16 (48) | 15 (42) | 16 (46) | 20 (65) |
| KMC** | 5 (15) | 19 (53) | 16 (46) | 1 (3) |
| Parental contact** | 2 (6) | 15 (42) | 7 (20) | 16 (52) |
| Nesting/positioning** | 1 (3) | 21 (58) | 16 (46) | 0 (0) |
| Soft touch** | 4 (12) | 16 (44) | 8 (23) | 8 (23) |
| Reduce light** | 2 (6) | 12 (33) | 6 (17) | 3 (10) |
| Reduce noise | 3 (9) | 9 (25) | 4 (11) | 2 (6) |
| Anything done in the unit to protect babies sleep? | | | | |
| No** | 21 (64) | 2 (6) | 2 (6) | 17 (55) |
| Nesting** | 5 (15) | 25 (69) | 21 (60) | 0 (0) |
| Dark/quiet periods** | 0 (0) | 19 (53) | 13 (37) | 8 (26) |
| Incubator covers * | 0 (0) | 10 (28) | 11 (31) | 0 (0) |
| Do cares/procedure when awake** | 0 (0) | 4 (11) | 2 (6) | 4 (13) |
| Change/comfortable position** | 2 (6) | 8 (242) | 0 (0) | 0 (0) |
| Clustered cares** | 1 (3) | 4 (11) | 3 (9) | 0 (0) |
| Anything done to in the unit to protect babies from light | | | | |
| No | 17 (52) | 2 (6) | 1 (3) | 2 (6) |
| Shaded windows/drawing curtains** | 8 (24) | 16 (44) | 1 (3) | 26 (84) |
| Eye masks for phototherapy** | 6 (18) | 30 (83) | 3 (9) | 12 (39) |
| Cover incubators** | 4 (12) | 10 (28) | 31 (89) | 2 (7) |
|  | 2 (6) | 28 (78) | (3)9 | 12 (39) |
| Anything done in the unit to protect babies from sound? | | | | |
| No | 31 (94) | 8 (22) | 32 (91) | 19 (61) |
| Turn off/limit alarms** | 2 (6) | 28 (78) | 0 (0) | 0(0) |
| No radio** | 0 (0) | 20 (56) | 0 (0) | 0 (0) |
| Talk softly** | 0 (0) | 11 (31) | 0 (0) | 1 (3) |
| Low or no volume on phones** | 0 (0) | 9 (25) | 0 (0) | 2 (6) |
| Trained in KMC** | 18 (55) | 32 (89) | 12 (34) | 24 (77) |
| How often should KMC be done in a 24 hour period? ** | | | | |
| As much as possible | 5 (15) | 12 (33) | 1 (3) | 6 (19) |
| 7-12 hours | 8 (24) | 9 (25) | 31 (89) | 18 (58) |
| 0-6 hours | 15 (45) | 10 (28) | 1 (3) | 3 (10) |
| Don’t know | 3 (6) | 3 (8) | 1 (3) | 4 (13) |
| What are the benefits of KMC? |  |  |  |  |
| Improves attachment/bonding | 23 (70) | 32 (89) | 31 (89) | 27 (87) |
| Decreases hypothermia** | 28 (85) | 34 (94) | 19 (54) | 25 (81) |
| Helps brain development** | 7 (21) | 28 (78) | 25 (71) | 3 (10) |
| Increases breastfeeding** | 7 (21) | 20 (56) | 23 (66) | 7 (23) |
| Decreases stress in the baby** | 1 (3) | 27 (75) | 5 (14) | 9 (29) |
| Decreases stress in the mother** | 1 (3) | 22 (61) | 13 (37) | 3 (10) |
| Reduces mortality/infection | 3 (9) | 9 (25) | 4 (11) | 5 (16) |
| Improves weight gain* | 6 (18) | 8 (22) | 4 (11) | 0 (0) |
| Has KMC been done in the unit in the last 7 days** | 30 (91) | 36 (100) | 34 (97) | 19 (61) |
| Do most parents practice KMC in this facility** | 25 (76) | 35 (97) | 33 (94) | 18 (58) |
| **Knowledge and practice of family care** | | | | |
| What are the benefits of families helping to care for their babies in the unit | | | | |
| Reduces staff workload** | 7 (21) | 18 (50) | 7 (20) | 19 (61) |
| Increased attachment/bonding** | 6 (18) | 18 (50) | 16 (46) | 20 (65) |
| Reduced parental stress** | 13 (39) | 28 (78) | 29 (83) | 20 (65) |
| Increases breastfeeding | 6 (18) | 13 (36) | 7 (20) | 11 (35) |
| Reduces babies stress/crying** | 6 (18) | 24 (67) | (4) 11 | 15 (48) |
| Increases parental skills** | 9 (27) | 18 (50) | 4 (11) | 6 (19) |
| Improves communication** | 9 (27) | 16 (44) | 3 (9) | 1 (3) |
| What are the negatives of families helping to care for babies in the unit | | | | |
| Infection risk | 21 (64) | 22 (61) | 29 (83) | 24 (77) |
| Poor handling of babies** | 11 (33) | 20 (56) | 5 (14) | 14 (45) |
| Crowding** | 4 (12) | 16 (44) | 16 (46) | 18 (58) |
| Bothering staff/interfering with care** | 2 (6) | 17 (47) | 5 (14) | 11 (35) |
| What role do mothers play in the care of their babies in the unit | | | | |
| Feed baby | 30 (91) | 35 (97) | 33 (94) | 31 (100) |
| Change nappies** | 19 (58) | 32 (89) | 17 (49) | 26 (84) |
| Wash clothes** | 5 (15) | 24 (67) | 16 (46) | 20 (65) |
| Clean e.g. incubator or cot** | 14 (42) | 6 (17) | 3 (9) | 11 (35) |
| Bath baby** | 3 (9) | 10 (28) | 0 (0) | 14 (45) |
| KMC | 3 (9) | 5 (14) | 6 (17) | 1 (3) |
| Check babies** | 5 (15) | 4 (11) | 2 (6) | 10 (32) |
| Comfort/emotionally support baby | 2 (6) | 2 (6) | 2 (6) | 1 (3) |
| What information is given to parents | | | | |
| Status of baby** | 19 (58) | 34 (94) | 35 (100) | 23 (74) |
| Feeding/expressing** | 19 (58) | 31 (86) | 7 (20) | 22 (71) |
| Hygiene** | 12 (36) | 28 (78) | 7 (20) | 12 (39) |
| Visiting times** | 18 (55) | 24 (67) | 2 (6) | 7 (23) |
| Protocols/rules | 0 (0) | 7 (19) | 3 (9) | 5 (16) |
| **Knowledge of establishing and maintaining breastmilk supply** | | | | |
| When should the mother of a pre-term start expressing milk?** | | | | |
| Immediately/within 6 hours | 8 (24) | 24 (67) | 12 (34) | 11 (35) |
| Day1-3 | 18 (55) | 1 (3) | 16 (46) | 12 (39) |
| >Day 3 | 2 (6) | 0 (0) | 0 (0) | 1 (3) |
| Depends on babies condition | 4 (12) | 9 (25) | 4 (11) | 6 (19) |
| How many times in a 24 hour period should a mother of a pre-term express milk** | | | | |
| 1-2 times | 7 (21) | 0 (0) | 0 (0) | 0 (0) |
| 3-4 times | 5 (15) | (3) 8 | 3 (9) | 1 (3) |
| 5-6 times | 1 (3) | 0 (0) | 0 (0) | 0 (0) |
| 7 or more | 18 (55) | 33 (92) | 28 (80) | 30 (97) |
| What can be done to help a mother stimulate her milk supply | | | | |
| Drink water/fluids | 16 (48) | 24 (67) | 20 (57) | 22 (71) |
| Breast massage** | 9 (27) | 21 (58) | 15 (43) | 18 (58) |
| Express/breastfeed often** | 6 (18) | 22 (61) | 5 (14) | 10 (32) |
| Express early** | 2 (6) | 19 (53) | 3 (9) | 8 (26) |
| KMC/contact with baby** | 0 (0) | 9 (25) | 12 (34) | 4 (13) |
| Reduce stress | 6 (18) | 7 (19) | 8 (23) | 1 (3) |
| Adequate diet | 3 (9) | 8 (22) | 5 (14) | 3 (10) |
| Are mothers provided with any equipment or containers for expressing | | | | |
| No** | 19 (58) | 0 (0) | 0 (0) | 17 (55) |
| Bowls/cups** | 11 (30) | 24 (67) | 18 (51) | 13 (42) |
| Bottles** | 2 (6) | 28 (78) | 32 (91) | 0 (0) |
| Syringes** | 1 (3) | 33 (92) | 0 (0) | 5 (16) |
| What are the most frequent ways equipment and containers for expressing are cleaned | | | | |
| Mother washes** | 24 (73) | 34 (94) | 1 (3) | 28 (90) |
| Staff washes** | 0 (0) | 29 (81) | 30 (86) | 0 (0) |
| Milton tablets** | 0 (0) | 24 (67) | 0 (0) | 0 (0) |
| Sterilized** | 0 (0) | 1 (3) | 14 (40) | 1 (3) |
